# Supplementary material for: Digital Alchemy: The Rise of Machine and Deep Learning in Small-Molecule Drug Discovery
Source: Int J Mol Sci. 2025 Jul 16;26(14):6807. doi: 10.3390/ijms26146807 (PMC12295739; doi:10.3390/ijms26146807)
Supplement: Supplementary file 1 [file ijms-26-06807-s001.zip › ijms-3736020-supplementary.pdf]

Int. J. Mol. Sci. **2025**

<Supplementary Material>

Digital Alchemy: The rise of Machine and Deep Learning in Small-Molecule Drug Discovery

Abdul Manan[1], Eunhye Baek[2], Sidra Ilyas[3*] and Donghun Lee [3*]

[1]Department of Molecular Science and Technology, Ajou University, Suwon 16499, Republic of Korea; Abdul Manan (mananriaz012@gmail.com)

[2] RexSoft Inc., 1 Gwanak-ro, Gwanak-gu, Seoul 08826, Republic of Korea; Eunhye Baek (1st-balloon@hanmail.net)

[3] Department of Herbal Pharmacology, College of Korean Medicine, Gachon University, 1342 Seong-namdae-ro, Sujeong-gu, Seongnam-si, 13120, Korea; Sidra Ilyas (sidrailyas6@gachon.ac.kr), Donghun Lee (dlee@gachon.ac.kr)

[*]Correspondence: sidrailyas6@gachon.ac.kr (S.I.); dlee@gachon.ac.kr (D.L.)

Supplementary Table S1: Comparative overview of deep learning (DL) architectures for drug design.

Aspect	ANN	DNN	CNN	RNN	GAN	VAE	GNN	RL
Mechanism	Fully connected layers	Deep ANN with multiple hidden layers	Convolutional and pooling layers	Recurrent connections with memory across time steps	Generator–Discriminator adversarial training	Probabilistic encoder-decoder with latent space	Message passing or attention on molecular graphs	Agent-environment interaction with reward-based learning
Input Representation	Feature vectors (e.g., descriptors)	High-dimensional vectors	Gridded/local data (e.g., images, 3D molecular grids)	Ordered sequences (e.g., SMILES, reactions, time series)	Latent vectors (optionally conditioned on properties)	Latent vectors from learned distributions	Molecular graphs (nodes = atoms, edges = bonds)	SMILES, molecular graphs, fragment-based assemblies
Strengths	Simple, fast, good baseline	Learns hierarchical abstractions	Spatial pattern recognition, translation invariance	Captures temporal/sequential dependencies	Generates realistic and novel molecules, diverse output generation	Smooth interpolation in latent space, controllable generation	Incorporates molecular topology, interpretable, state-of-the-art models	Goal-directed learning, supports multi-objective optimization
Applications	QSAR modeling, property prediction	Complex ADMET prediction, multi-omics integration	Binding site analysis, phenotypic screening	SMILES generation, reaction outcome prediction	Scaffold decoration, *de novo* generation, chemical space exploration	Latent space exploration, controlled *de novo* design	Property prediction, drug–target affinity, drug–drug interaction	Active learning, fragment-based generation, docking/ADMET optimization

Advantages	Easy to implement, minimal preprocessing	Captures nonlinear relationships	Local feature extraction, fewer parameters than FCs	Handles variable-length input, maintains sequence context	High-quality molecule generation, scaffold optimization	Easy property conditioning, explicit likelihood estimation	High accuracy, graph attention, suitable for structured data	Flexible with arbitrary rewards, adaptable to different design goals
Limitations	Poor performance on complex tasks	Needs large datasets, risk of overfitting	Needs grid-formatted input, high computational cost	Gradient vanishing/exploding, slower training	Instability, mode collapse, sensitive to hyperparameter tuning	Risk of limited diversity, may oversimplify latent prior	Sensitive to graph quality, resource intensive, data hungry	Sample inefficient, reward design critical, prone to local optima

Supplementary Table S2: Evolution and relationship of deep neural networks.

Model Type	Derived From	Key Idea / Feature	Primary Use Case	Notes / Subtypes
ANN	-	Basic feedforward neural network	General function approximation	Perceptron is a simple form
DNN	ANN	Multiple hidden layers	Complex pattern learning	Needs careful training (activation, init)
CNN	DNN	Convolution + pooling for spatial data	Image classification, vision tasks	LeNet, AlexNet, VGG, ResNet, EfficientNet
RNN	DNN	Loops for sequential data	Time series, text, speech	Vanilla RNNs → LSTM → GRU
AE	DNN	Encoder-decoder for reconstruction	Feature extraction, denoising	Sparse AE, Denoising AE, Variational AE
GAN	DNN (dual models)	Generator vs Discriminator adversarial game	Image generation, synthesis	StyleGAN, CycleGAN
Attention	RNN (Seq2Seq)	Focus on relevant input parts (soft attention)	Translation, alignment	Paved the way for Transformers
Transformer	Attention	Self-attention + feedforward, no recurrence	NLP, code, audio, vision (ViT)	BERT, GPT, T5, BART, etc.
ViT	Transformer	Transformer over image patches	Vision tasks, classification	Competes with CNNs
GNN	Inspired by CNN/RNN	Neural nets over graph structures	Graphs, social networks, molecules	GCN, GAT, GraphSAGE
